# Cell Viability of Porous Poly(d,l-lactic acid)/Vertically Aligned Carbon Nanotubes/Nanohydroxyapatite Scaffolds for Osteochondral Tissue Engineering

**DOI:** 10.3390/ma12060849

**Published:** 2019-03-13

**Authors:** Thiago Domingues Stocco, Eliane Antonioli, Conceição de Maria Vaz Elias, Bruno Vinícius Manzolli Rodrigues, Idália Aparecida Waltrick de Brito Siqueira, Mario Ferretti, Fernanda Roberta Marciano, Anderson Oliveira Lobo

**Affiliations:** 1 Faculty of Medical Sciences, State University of Campinas, São Paulo 13083-887, Brazil; tdstocco@live.com; 2 Faculty of Physiotherapy, University of Santo Amaro, São Paulo 04829-300, Brazil; 3 Hospital Israelita Albert Einstein, São Paulo 05652-000, Brazil; elianeantonioli@gmail.com (E.A.); Mario.Ferretti@einstein.br (M.F.); 4 Scientifical and Technological Institute, Brasil University, São Paulo 08230-030, Brazil; conceicaovazenf@hotmail.com (C.d.M.V.E.); bruno.manzolli@gmail.com (B.V.M.R.); femarciano@gmail.com (F.R.M.); 5 Institute of Science and Technology, Federal University of São Paulo, São José dos Campos, São Paulo 12231-280, Brazil; idaliasiqueira@yahoo.com.br; 6 LIMAV-Interdisciplinary Laboratory for Advanced Materials, UFPI-Federal University of Piauí, Teresina 64049-550, Piauí, Brazil

**Keywords:** osteochondral regeneration, nanocomposites, porous scaffolds, carbon nanotubes, PDLLA, hydroxyapatite, chondrocyte

## Abstract

Treatment of articular cartilage lesions remains an important challenge. Frequently the bone located below the cartilage is also damaged, resulting in defects known as osteochondral lesions. Tissue engineering has emerged as a potential approach to treat cartilage and osteochondral defects. The principal challenge of osteochondral tissue engineering is to create a scaffold with potential to regenerate both cartilage and the subchondral bone involved, considering the intrinsic properties of each tissue. Recent nanocomposites based on the incorporation of nanoscale fillers into polymer matrix have shown promising results for the treatment of osteochondral defects. In this present study, it was performed using the recently developed methodologies (electrodeposition and immersion in simulated body fluid) to obtain porous superhydrophilic poly(d,l-lactic acid)/vertically aligned carbon nanotubes/nanohydroxyapatite (PDLLA/VACNT-O:nHAp) nanocomposite scaffolds, to analyze cell behavior and gene expression of chondrocytes, and then assess the applicability of this nanobiomaterial for osteochondral regenerative medicine. The results demonstrate that PDLLA/VACNT-O:nHAp nanocomposite supports chondrocytes adhesion and decreases type I Collagen mRNA expression. Therefore, these findings suggest the possibility of novel nanobiomaterial as a scaffold for osteochondral tissue engineering applications.

## 1. Introduction

The regeneration of articular cartilage injuries and defects remains as one of the most important challenges for orthopedic surgeons and researchers [1,2]. Due to its avascular nature, cartilage tissue has poor intrinsic repair and limited regenerative capacity. Thus, damages in this tissue are normally irreparable and difficult to treat [3].

The causes of cartilage lesions are usually multifactorial and occur as a result of acute and/or repetitive trauma, chronic degenerative diseases and aging [4]. The bone located below the cartilage is also frequently damaged, which result in defects known as osteochondral lesions [5]. Osteochondral defects are usually symptomatic and, if untreated, lead to the development of osteoarthritis, the most common worldwide joint disease, affecting a quarter of the world adult population. This disease represents the second greatest cause of physical disability and has a deep socioeconomic impact [2,6,7,8]. Thus, it is possible to understand that the clinical implication and societal impact of novel treatment procedures can be huge.

The distinct characteristics of the articular cartilage and subchondral bone, such as biochemical composition, regeneration capacity, mechanical properties, and the complexity of the bone-cartilage interface, impair the successful treatment of these injuries [2,9,10]. Currently, the available therapeutic options for osteochondral defects include bone marrow stimulation techniques, osteochondral allografts, debridement and microfracture. Along with debridement and microfracture, the techniques for bone marrow stimulation produce unsatisfactory results in long-term periods and are only palliative, not curative [4,11,12,13]. Osteochondral allografts have limitations due to transplant rejection, risk of disease transmission, limited availability of the donor tissues, and exhibit a high failure rate [4,11,14,15]. Therefore, there is an urgent need for the development of effective methods and potential therapies for treating osteochondral defects.

In this context, tissue engineering has emerged as a promising approach to treat cartilage and osteochondral defects [3,4]. However, the success of this strategy is closely linked to the development of adequate biomaterials that are able to encourage, support and guide tissue growth. Ideally, a scaffold must be biocompatible, bioresorbable and present a degradation rate that corresponds to the rate of the newly formed tissue. Furthermore, an ideal scaffold should be porous, which allows for nutrient diffusion and transport, and should have similar physical, chemical and mechanical properties to the native osteochondral tissue, being able to promote cell adhesion, proliferation and differentiation [5,9,16].

Synthetic biodegradable polymers, such as poly(lactic acid) (PLA), poly(d,l-lactic acid) (PDLLA), polycaprolactone (PCL) and poly(glycolic acid) (PGA) have been investigated to construct scaffolds for application in cartilage tissue engineering [17,18,19,20]. Recombinant Human Bone Morphogenetic Protein-2 (rhBMP-2), marine collagen, titanium micromesh and bioceramic scaffolds, such as the ones from hydroxyapatite (HA), calcium phosphate and bioglass, have also been extensively investigated for bone reconstruction and regeneration [21,22,23,24,25]. Nevertheless, the main challenge of osteochondral tissue engineering is to create a scaffold with potential to regenerate both cartilage and the subchondral bone, taking into account the specific intrinsic properties of each tissue [26,27]. From this perspective, scaffolds based on a single component, i.e., bioceramic or polymer, have shown a series of limitations. Accordingly, composites combining both polymer and bioceramics have been extensively explored [9,28,29,30,31].

Recent studies on nanocomposites based on polymers and nanoparticles have shown promising results for the treatment of osteochondral defects [32,33,34,35,36]. The incorporation of nanoscale fillers, such as carbon nanotubes (CNT) and nanohydroxyapatite (nHAp) into polymer matrices has allowed for the improvement of the chemical, physical and biological properties of the final scaffold [37,38,39,40]. 

In a previous study in the research group [41], the production of a novel porous superhydrophilic PDLLA/CNT/nHAp nanocomposite by two different methodologies was explored. This scaffold showed to be able to mimic the immature bone and induced bone remodeling. The results also proved that this novel nanomaterial promoted bioactivity with no traces of cytotoxicity. However, when it comes to the application of this material as scaffold to osteochondral lesions some aspects remain underexplored, mainly the chondrocytes behavior and their potential for regeneration of cartilaginous tissue.

Thus, herein was proposed the evaluation of porous PDLLA/CNT/nHAp scaffolds obtained from recently developed methodologies [41], in order to analyze their cell behavior and gene expression of chondrocytes. Therefore, it is aimed at to assess the applicability of this nanobiomaterial for osteochondral regenerative medicine.

## 2. Materials and Methods

### 2.1. Preparation of Superhydrophilic Vertically Aligned Multi-Walled Carbon Nanotubes Films (VAMWCNT-O)

Vertically aligned multi-walled carbon nanotubes (VAMWCNT) were produced using a microwave plasma chamber at 2.45 GHz (MWCVD), as described elsewhere [42]. Briefly, titanium (Ti) squares (10 mm), covered by a thin Fe layer (10 nm) deposited by an e-beam evaporator, were used as substrates. A pre-treatment was carried out for 5 min in N_2_/H_2_ (10/90 cm^3^ (STP) min^−1^) plasma, at 760 °C, in order to generate the catalyst for VAMWCNT growth. After this pre-treatment, CH_4_ (14 cm^3^ (STP) min^−1^) was inserted into the chamber for VAMWCNT nucleation. The pressure and temperature were kept around 800 °C and 30 Torr, respectively. Next, oxygen was incorporated by using a pulsed-direct current plasma reactor. Briefly, an oxygen flow rate of 1 cm^3^ (STP) min^−1^, at 85 mTorr, 700 V, frequency of 20 kHz and a time of treatment of 120 s was used. After oxygen plasma treatment, the samples were further referred as VAMWCNT-O.

### 2.2. Electrodeposition of nHA Crystals on the VAMWCNT-O (nHAp1)

nHAp crystals were electrodeposited onto the VAMWCNT-O films as previously reported [43], using Ca(NO_3_)_2_·4H_2_O and (NH_4_)·2HPO_4_ electrolytes (pH = 4.8) at 0.042 mol·L^−1^ and 0.025 mol·L^−1^, respectively. For the electrochemical measurements, a three-electrode cell apparatus coupled to an Autolab PGSTAT 128N equipment was used. VAMWCNT-O films were used as the working electrode. VAMWCNT-O films had a geometric area of 0.27 cm^2^ (contact area with the electrolytic solution). A platinum coil wire was used as an auxiliary electrode, while an Ag/AgCl electrode was used as reference electrode. The production of the nHAp crystals occurred by simply applying a constant potential of −2.0 V for 30 min, and the solution temperature was kept at 70 °C in constant stirring.

### 2.3. Deposition of nHA Crystals on the VAMWCNT-O Using Simulated Body Fluid (nHAp2)

The VAMWCNT-O samples were immersed into a solution of simulated body fluid (SBF) (5×) (pH = 7.4) for the deposition of nHAp crystals. SBF solution (5×) was prepared using salts at different concentrations, as follows: NaCl: 733.5 mM, MgCl_2_·6H_2_O: 7.5 mM, CaCl_2_·2H_2_O: 12.5 mM, Na_2_HPO_4_·2H_2_O: 5.0 mM and NaHCO_3_: 21.0 mM [44,45].

The VAMWCNT-O samples were placed in plastic tubes and exposed to UV light in a biosafety chamber (BioProtector-12 Plus VECO, Campinas, Brazil) for 30 min. Next, 13 mL of SBF solution was added to the plastic tubes, which was stored in a refrigerated incubator (CT-712-R, Cientec, Porto Alegre, Brazil) under constant stirring (75 rpm at 36.5 °C) for 14 days. Finally, the samples were washed with deionized water (60 °C) and dried in an incubator (SP400, SPLABOR, Presidente Prudente Brazil) at 50 °C for 1 h [45,46].

### 2.4. Production of Porous PDLLA/VAMWCNT-O:nHAp Scaffolds

First, the nanoparticles of nHAp1 and nHAp2 from their Ti substrates were removed and dispersed in chloroform (0.3 wt%) under sonication (3 min, 1200 J mL^−1^). Thereafter, PDLLA (copolymer of l-lactide and d-lactide, 96/04, PLD-9655, Purasorb^®^) was dissolved in the VAMWCNT-O:nHAp/chloroform solution (10% w/v) (nHA1 and nHA2) under mechanical stirring for 120 min [47]. Subsequently, the solutions were placed into molds (0.5 mm in diameter) and kept under the following conditions: 80% controlled humidity (~80%) and room temperature. Next, a simple and fast functionalization to obtain superhydrophilicity in the porous polymer membranes was performed using a pulsed-direct current plasma reactor with an oxygen flow rate of 1 cm^3^ (STP) min^−1^, at a pressure of 85 mTorr, 700 V, at a repetition rate of 20 kHz [41]. A PDLLA membrane without any treatment or nanoparticles was prepared as a control. 

### 2.5. Characterization of Porous PDLLA/VAMWCNT-O:nHAp Membranes

Scanning electron microscopes (SEM) were used to evaluate the composition and morphology of the porous membranes. Before analysis, PDLLA, PDLLA/VAMWCNT-O:nHAp1 and PDLLA/VAMWCNT-O:nHAp2 were coated with a thin gold layer to improve the image acquisition. After, a SEM (JEOL JSM 5610 VPI, Tokyo, Japan) was used for the magnifications ranging between 100× and 15,000×, and a high-resolution SEM (Field emission Gun, FEG-SEM JSM 6330F, JEOL, Tokyo, Japan) was used to obtain magnifications ranging between 10,000× and 100,000×.

### 2.6. Cell Culture Experiments and Analysis

Healthy human chondrocyte cultures were isolated from normal articular cartilage of three young adults (age range: 26–36) undergoing ACL (anterior cruciate ligament) surgery. Small slices of cartilage were minced and digested overnight in 0.25% type I collagenase (Sigma-Aldrich, St. Louis, MO, USA), at 37 °C. The cells were then seeded onto tissue culture flasks, for expansion in monolayer, and kept as sub-confluent monolayers in growth medium, Dulbecco’s modified Eagle’s medium (DMEM) supplemented with 1.5 mL glutamine, 10% fetal bovine serum, and 100 units/mL penicillin–streptomycin (Gibco-Invitrogen, MA, USA). The incubation occurred in a humidifier at 37 °C and 5% CO_2_. The medium was replaced 3 times a week until cells reached 80% of confluence. Then, the cells were washed with phosphate buffer saline (PBS), removed with trypsin/ ethylenediaminetetra-acetic acid solution, and replaced at the same density until the third passage. The study was conducted in full accordance with local ethics guidelines, approved by the Ethics Committee (CEP/Hospital Israelita Albert Einstein nº10/1268; CAAE 0006.0.028.000-10), and cartilage samples were collected after obtaining written informed consent of the donors.

### 2.7. Cell Adhesion and Viability of PDLLA/VAMWCNT-O:nHAp Membranes

PDLLA, PDLLA/VAMWCNT-O:nHAp1 and PDLLA/VAMWCNT-O:nHAp2 membranes were cut in pieces of 0.5 cm (in diameter) and placed in a 24-well plate. Then, all samples were sterilized by UV light exposition for 2 h. Human chondrocytes were seeded in these membranes at a concentration of 5 × 10^5^ cells/mL (50 µL of cell suspension) and incubated for five days. Chondrocytes cultivated on plastic and PDLLA were used as the control group.

To determine the cell adhesion and viability, the chondrocyte density in each membrane was observed. After five days in culture, attached cells on membranes were fixed with 4% paraformaldehyde solution for 15 min, washed three times in PBS buffer and stained with a 0.05% crystal violet solution for 5 min, washed with PBS twice and then analyzed by optical microscope (FSX 100, Olympus, Tokyo, Japan). Cells were counted using ImageJ software (NIH, Bethesda, MD, USA).

The morphology of chondrocytes on the different membranes was evaluated by SEM (Zeiss EVO MA 10 microscope, Oberkochen, German) after five days in the culture. Cells were fixed with 3% glutaraldehyde/0.1 M sodium cacodylate buffer for 1 h, followed by a dehydration step with 10 min of incubation in a graded ethanol solution series (30%, 50%, 70%, 95%, and 100%). Next, a drying stage with a 1:1 solution of ethanol/hexamethyldisilazane at room temperature was performed. Samples were coated with a thin gold film to improve image acquisition. 

### 2.8. Cytotoxicity Assay

PDLLA, PDLLA/VAMWCNT-O:nHAp1 and PDLLA/VAMWCNT-O:nHAp2 scaffold-seeded cell were reacted with a lactate dehydrogenase (LDH) assay for the cytotoxicity test. After 24 h of culture, each group was analyzed by the LDH colorimetric assay kit (ab102526, Abcam) following the manufacturer’s instructions. In brief, 50 µL of supernatant of each group and LDH positive control was mixed with the reaction mix (substrate), incubated at 37 °C for 30 min. The result was determined by the spectrophotometer at 450 nm. Total LDH was expressed as [OD_sample_−OD_blank_]. Cells cultivated on plastic were used as the control. Results shown are representative of at least two independent experiments performed in triplicate and are expressed as mean ± SD (error bars) of three replicates.

### 2.9. Gene Expression Analysis

Relative quantification of mRNA expression of Sox-9, Aggrecan, MMP3, Type I and II Collagen was performed using Polymerase chain reaction quantitative real time (qRT-PCR). Total RNA from chondrocytes cultured in PDLLA, PDLLA/VAMWCNT-O:nHAp1, and PDLLA/VAMWCNT-O:nHAp2 membranes, for five days, was extracted by RNeasy kit (QIAGEN, Hilden, Germany) and reverse transcriptase reaction (QuantiTect Reverse Transcription kit, QIAGEN) was performed. qRT-PCR was carried out using an ABI7500 thermocycler (Applied Biosystems, Foster City, CA, USA) and the Maxima SYBR Green qPCR Master Mix (Thermo Fisher Scientific, Waltham, MA, USA), according to manufacturer’s recommendations. Primer sequences and PCR parameters were provided upon request. Sequences were detailed on Table 1. Expression of target genes was normalized by β-actin mRNA levels measured concurrently. The level of expression was then calculated as 2-ΔΔCt and expressed as the mean. All quantitative RT-qPCR results were representative of at least two independent experiments, each with three technical replicates and are expressed as mean ± SD (error bars).

### 2.10. Statistics Analysis

Data were expressed as mean ± SD. Statistical significance was determined by a two-tailed unpaired t test, one-way ANOVA in Graph Pad Prism 6^®^ software (GraphPad, CA, USA). Significance was determined at *p* < 0.05.

## 3. Results and Discussion

**Figure 1:**
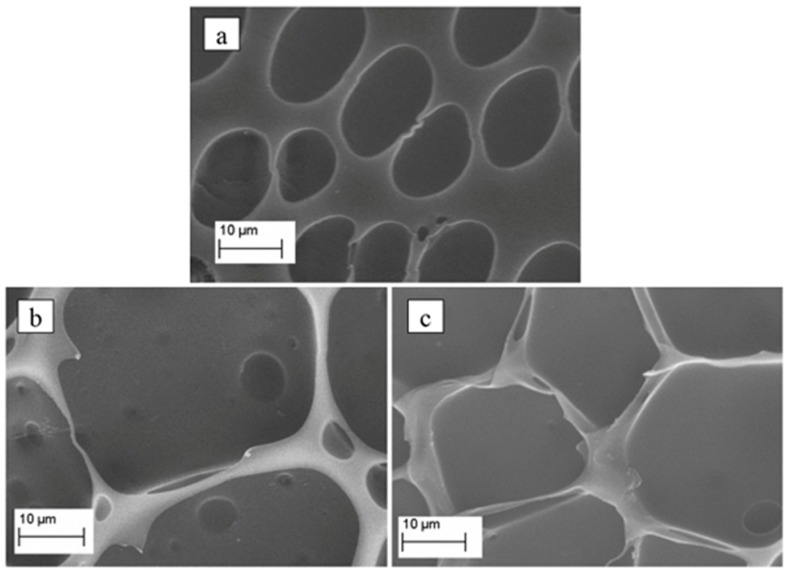
Micrographs of porous (**a**) PDLLA, (**b**) PDLLA/VAMWCNT-O:nHAp1, and (**c**) PDLLA/VAMWCNT-O:nHAp2 membranes.

Figure 1 shows micrographs of the produced porous PDLLA (Figure 1a), PDLLA/VAMWCNT-O:nHAp1 (Figure 1b), and PDLLA/VAMWCNT-O:nHAp2 (Figure 1c) membranes. The differences can be clearly observed as regards to the surface texture/morphology after the nanoparticles were incorporated. Figure 1a illustrates the smaller pores (~11–18 µm) on the surface of PDLLA. Conversely, the loading of both nHAp1 and nHAp2 led to larger pores on the surface (~27–39 µm, Figure 1b,c, respectively). 

Different composites using PDLLA have been described as biocompatible support materials for cell culture, focusing especially on their applicability in bone tissue engineering [41,47,48]. Moreover, many studies using PDLLA/HAp composites have been investigated employing different processes [49,50,51,52,53]. The incorporation of HAp and/or CNT in PDLLA polymer matrices is an important strategy to induce cell differentiation and ossification. Recent studies in the research group showed the biocompatibility of nHAp obtained by the method of immersion in SBF and also by electrodeposition [46,54]. 

**Figure 2:**
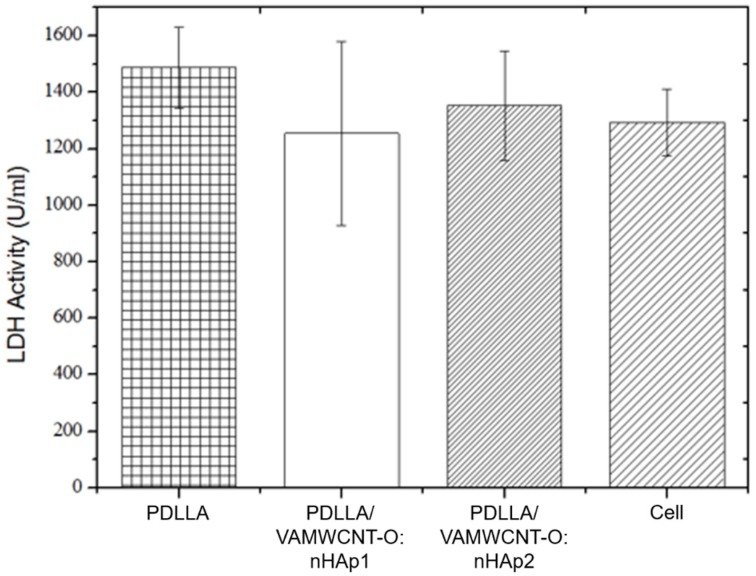
Cell viability of human chondrocytes on PDLLA, PDLLA/VAMWCNT-O:nHAp1, PDLLA/VAMWCNT-O:nHAp2, Cell (Plastic), (N = 3).

Cell viability is one of the preliminary tests conducted to demonstrate the possible cytotoxicity effects of a biomaterial candidate to be used as scaffold. The LDH test can show death and cell lysis after 24 h of culture and in a single incubation time (5 days); it is a biocompatibility test widely used for basic research on biomaterials, mainly involving osteochondral applications. Accordingly, the analysis of lactate dehydrogenase (LDH) showed that all membranes were non-toxic to human chondrocytes (Figure 2). This result illustrated that the different combinations of PDLLA and hydroxyapatite can alter the viability of human chondrocytes similar to that of the control (cells cultivated at plastic). 

**Figure 3:**
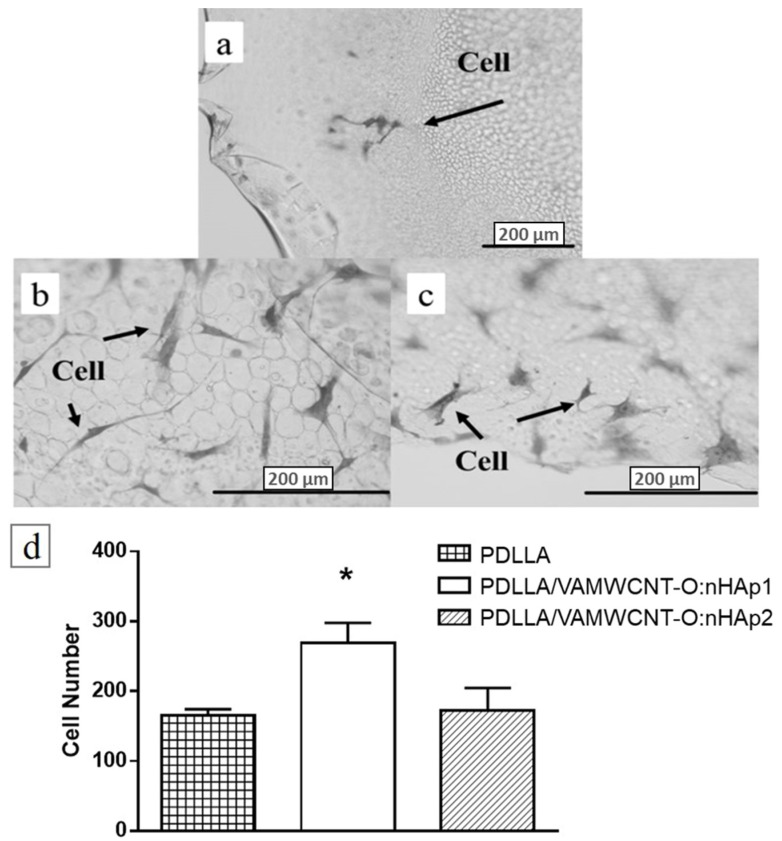
Optical microscopy of the human chondrocytes adhered on (**a**) PDLLA, (**b**) PDLLA/VAMWCNT-O:nHAp1, (**c**) PDLLA/VAMWCNT-O:nHAp2 membranes, (**d**) cell number of chondrocytes attached (N = 3). ANOVA one way with Post-test Tukey’s multiple comparisons test (* *p* < 0.05, PDLLA/VAMWCNT-O:nHAp1 vs. PDLLA and PDLLA/VAMWCNT-O:nHAp1 vs. PDLLA/VAMWCNT-O:nHAp2).

In this study, it is possible to verify the performance of the chondrocytes on the PDLLA/VAMWCNT-O:nHAp membranes. After five days in culture, PDLLA/VAMWCNT-O:nHAp membranes showed a higher number of chondrocytes attached than PDLLA membranes (Figure 3). Five fields from each group were recorded and performed in triplicate. This effect may be explained by a result of the highest porosity of PDLLA/VAMWCNT-O:nHAp membranes, suggesting that the presence of VAMWCNT-O combined by nHAp using SBF, is able to improve chondrocytes survival.

**Figure 4:**
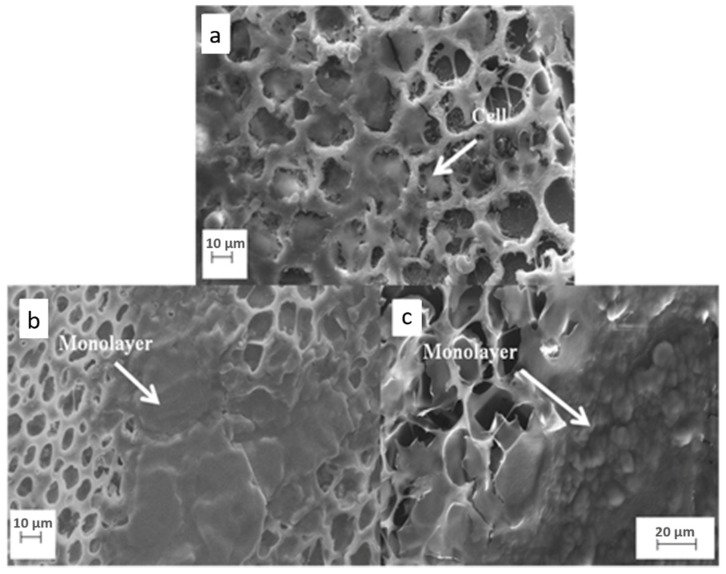
SEM Micrographs of human chondrocytes adhesion on (**a**) PDLLA, (**b**) PDLLA/VAMWCNT-O:nHAp1, and (**c**) PDLLA/VAMWCNT-O:nHAp2 scaffolds.

SEM analysis showed the chondrocytes morphology cultivated in the different membranes. In porous PDLLA/VAMWCNT-O:nHAp1 and PDLLA/VAMWCNT-O:nHAp2 membranes, it can be observed that cells organized on the surface of porous membranes, occupied a large area of material coverage, confirming that these membranes promoted the survival of chondrocytes after five days in culture (Figure 4b,c). The chondrocytes protruded into the interior of membrane pores, occupying the full diameter and forming a cellular layer. 

**Figure 5:**
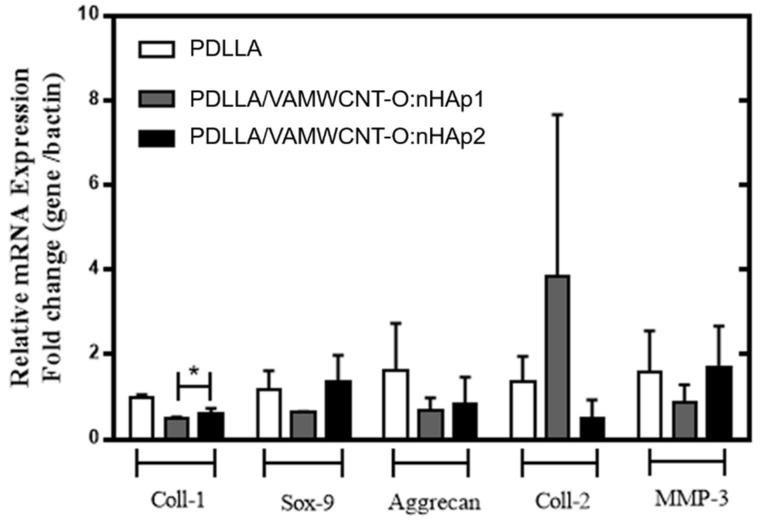
Gene expression of human chondrocytes on PDLLA, PDLLA/VAMWCNT-O:nHAp1, and PDLLA/VAMWCNT-O:nHAp2 membranes after five days in culture. The bar graphs show relative gene expression levels after normalization to beta-actin.

The expression of typical chondrogenic markers was evaluated by PCR analysis. Figure 5 shows the gene expression levels for type I Collagen, type II Collagen, Aggrecan, MMP-3 and Sox-9 of the chondrocytes cultivated on porous PDLLA, PDLLA/VAMWCNT-O:nHAp1, and PDLLA/VAMWCNT-O:nHAp2 membranes up to five days. 

The results suggests that chondrocytes cultivated on porous PDLLA/VAMWCNT-O:nHAp1 and PDLLA/VAMWCNT-O:nHAp2 membranes showed lower mRNA of Type I Collagen expression than chondrocytes cultivated on porous PDLLA membranes (control; *p* < 0.05). 

Conversely, the different PDLLA preparation did not alter the expression levels of the other genes analyzed. The absence of change in all sets of genes can be elucidated by a short period of time and a low number of samples analyzed. These results corroborate with other researches that reported that the detection of the type II Collagen and Aggrecan mRNA expression only occurs after 7–14 days of the cell culture on biomaterial [55,56]. Stenhamre et al. (2013) reported that the chondrocytes re-differentiation maintained fibroblastoid format after 7 days of culture when cultivated on 3D nanofibers mats [57]. Similar results were attained after 5 days of cultivation on porous membranes with incorporated nanoparticles present. The lower type I Collagen mRNA expression observed in chondrocytes cultivated on porous PDLLA/VAMWCNT-O:nHAp1 and PDLLA/VAMWCNT-O:nHAp2 membranes suggest that, that is the first step of re-differentiation state, shifting the microenvironment similar to health cartilage. 

In monolayer culture on a substrate, chondrocytes lose their spherical morphology and acquire a fibroblast-like format. This expansion causes a process of dedifferentiation characterized by decreased synthesis of type II collagen and increased synthesis of type I collagen [58,59,60]. The re-differentiation process, which promotes cartilage regeneration, depends on several factors such as time of culture, physical and chemical characteristics of the scaffold [57]. However, the controlled polymer degradation and the porosity to allow free exchange of body fluids, can promote the rescue to the spherical format and phenotype characteristic of chondrocytes [61]. 

The results showed that cells adhered on the porous membranes (Figure 3 and Figure 4), and do not present cytotoxic effects (Figure 2). However, the cells behave differently in porous PDLLA/VAMWCNT-O:nHAp membranes, the results showed an increase in adhesion and formation of monolayer cells in the PDLLA/VAMWCNT-O:nHAp membranes. It is probably due to the increase in porosity of surfaces, similar behavior has been previously described by Siqueira et al. (2015) in bone regeneration. Besides that, others researchers demonstrated the influence of surface wettability and increased porosity on cell adherence behavior and re-differentiation of chondrocytes because these surface properties improve the cell adhesion capacity [61,62,63,64,65,66,67].

This paper has shown that both the porous PDLLA/VAMWCNT-O:nHAp1 and PDLLA/VAMWCNT-O:nHAp2 membranes promote chondrocytes adhesion (Figure 3 and Figure 4), non-cytotoxic effects (Figure 2) and decrease the type I Collagen mRNA expression (Figure 5). Therefore, the clinical implication of this study was that since previously these nanobiomaterial had already demonstrated biocompatibility with osteoblasts [41], the findings are very encouraging for its use as scaffolds for the treatment of osteochondral defects. Moreover, this study contributes to the knowledge of the chondrocytes behavior on different biomaterials.

In the future, complementary studies, including increases in sample number, longer periods of time culture and an in vivo evaluation, will be performed before translation to clinical use.

## 4. Conclusions

The spreading, adhesion, and gene expression of human chondrocytes cultivated on two different compositions of membranes was performed. The porosity and roughness in PDLLA/VAMWCNT-O:nHAp were associated to partial hydrophobicity control after a simple and fast oxygen plasma etching due to oxygen groups attached on the surface. All these surface controls promote human chondrocytes adhesion, decrease type I Collagen mRNA expression and non-cytotoxic effects. However, although these results suggest porous PDLLA/VAMWCNT-O:nHAp1 and PDLLA/VAMWCNT-O:nHAp2 membranes as potential alternatives in osteochondral repair, additional in vivo assays will be necessary to fully elucidate the clinical implication of our observations.

## Figures and Tables

**Table 1:** Sequence of primers.

MMP-3	sense 5-ATTCCATGGAGCCAGGCTTTC-3′
	anti-sense 5′CATTTGGGTCAAACTCCACTGTG-3′
Type I Collagen	sense 5-CCGCCGCTTCACCTACAGC-3′
	antisense 5-TTTGTATTCAATCACTGTCTTGCC-3′
Type II Collagen	sense 5-CCGAATAGCAGGTTCACGTACA-3′
	antisense 5-CGATAACAGTCTTGCCCCACTT-3′
Aggrecan	sense 5-TTCAGTGGCCTACCAAGTGG-3′
	antisense 5-AGCCTGGGTTACAGATTCCA-3′
Sox-9	sense 5-TGCTAGAAGATGAGGCTTCTGG-3′
	antisense 5-GGCACTTTGTCCAGACCCA-3′
Beta-actin	sense 5′-GGCACCCAGCACAATGAAG-3′
	antisense 5′-CCGATCCACACGGAGTACTTG-3′
